# Pretreatment serum lactate level as a prognostic biomarker in patients undergoing supratentorial primary brain tumor resection

**DOI:** 10.18632/oncotarget.18891

**Published:** 2017-06-29

**Authors:** Chung-Chih Shih, Tzong-Shiun Lee, Fon-Yih Tsuang, Pei-Lin Lin, Ya-Jung Cheng, Hsiao-Liang Cheng, Chun-Yu Wu

**Affiliations:** ^1^ Department of Anesthesiology, National Taiwan University Hospital, Taipei, Taiwan; ^2^ Division of Neurosurgery, Department of Surgery, National Taiwan University Hospital, Taipei, Taiwan

**Keywords:** lactate, brain tumor, tumor marker, prognostic factor, glioma

## Abstract

**Introduction:**

Malignant primary brain tumors are one of the most aggressive cancers. Pretreatment serum nonneuronal biomarkers closely associated with postoperative outcomes are of high clinical relevance. The present study aimed to identify potential pretreatment serum biomarkers that may influence oncological outcomes in patients with primary brain tumors.

**Methods:**

A total of 74 patients undergoing supratentorial primary brain tumor resection were enrolled. Before tumor resection, serum neuronal biomarkers, namely neuron-specific enolase (NSE), S100β, and glial fibrillary acidic protein (GFAP), and serum nonneuronal biomarkers, namely neutrophil gelatinase-associated lipocalin (NGAL), lactate dehydrogenase (LDH), and lactate, were measured and associated postoperative oncological outcomes, including brain tumor grading, progression-free survival (PFS), and overall survival (OS), were compared.

**Results:**

Patients with high-grade brain tumors had significantly higher pretreatment serum lactate levels (*p* = 0.011). By contrast, other biomarkers were comparable between patients with high-grade and low-grade brain tumors. Receiver operating characteristic curve analysis of serum lactate levels yielded an area under the curve of 0.71 for differentiating between high-grade and low-grade brain tumors. Kaplan–Meier survival analysis revealed patients with high serum lactate levels (≧2.0 mmol/L) had shorter PFS and OS (*p* = 0.021 and *p* = 0.093, respectively). In a multiple regression model, only elevated serum lactate levels were associated with poor PFS and OS (*p* = 0.021 and *p* = 0.048, respectively).

**Conclusions:**

An elevated pretreatment serum lactate level is a prognostic biomarker of high-grade primary brain tumors and is significantly associated with poor PFS in patients with supratentorial brain tumors undergoing tumor resection. By contrast, other serum biomarkers are not significantly associated with oncological outcomes.

## INTRODUCTION

The oncological outcomes for brain tumors remain poor even after a successful tumor resection. Serum biomarkers, which represent brain tumor severity and are associated with therapy, may not only improve differential diagnosis but also facilitate clinical management of patients with brain tumors. Elevated levels of several serum biomarkers, including neuronal and nonneuronal biomarkers, have been reported in patients with brain tumors. Serum neuronal biomarkers, such as neuron-specific enolase (NSE) [1], S100β [2], and glial fibrillary acidic protein (GFAP) [3, 4], have been reported to be upregulated in patients with brain tumors caused by brain injuries. However, the association between serum neuronal biomarkers and oncological outcomes after tumor resection has yet to be sufficiently investigated in patients with brain tumors.

Elevated levels of nonneuronal biomarkers, including tissue and urinary neutrophil gelatinase-associated lipocalin (NGAL) and tissue lactate, have been reported in patients with brain tumors. NGAL is a member of the lipocalin protein family, and plays multifaceted roles in both physiological and pathological processes, such as inflammatory responses and cell proliferation. Higher tissue and urinary NGAL levels have been reported in patients with brain tumors [5, 6]. However, as a bridge between tissue and urinary biomarkers, serum NGAL levels have been investigated insufficiently in patients with brain tumors.

In a tumor microenvironment, lactate is produced by tumor cells and immune cells through anaerobic glycolysis [7] catalyzed by lactate dehydrogenase (LDH). Accordingly, higher serum lactate levels were reported in patients before brain tumor resection [8], and higher serum LDH was related to oncological outcomes in patients with solid tumors [9]. However, the prognostic value of pretreatment serum lactate levels has yet to be compared with that of other serum biomarkers, particularly neuronal biomarkers. Therefore, this study investigated the potential pretreatment prognostic value of serum neuronal and nonneuronal biomarkers, namely NSE, S100β, GFAP, NGAL, LDH, and lactate, in patients undergoing supratentorial primary brain tumor resection.

## RESULTS

**Figure 1:**
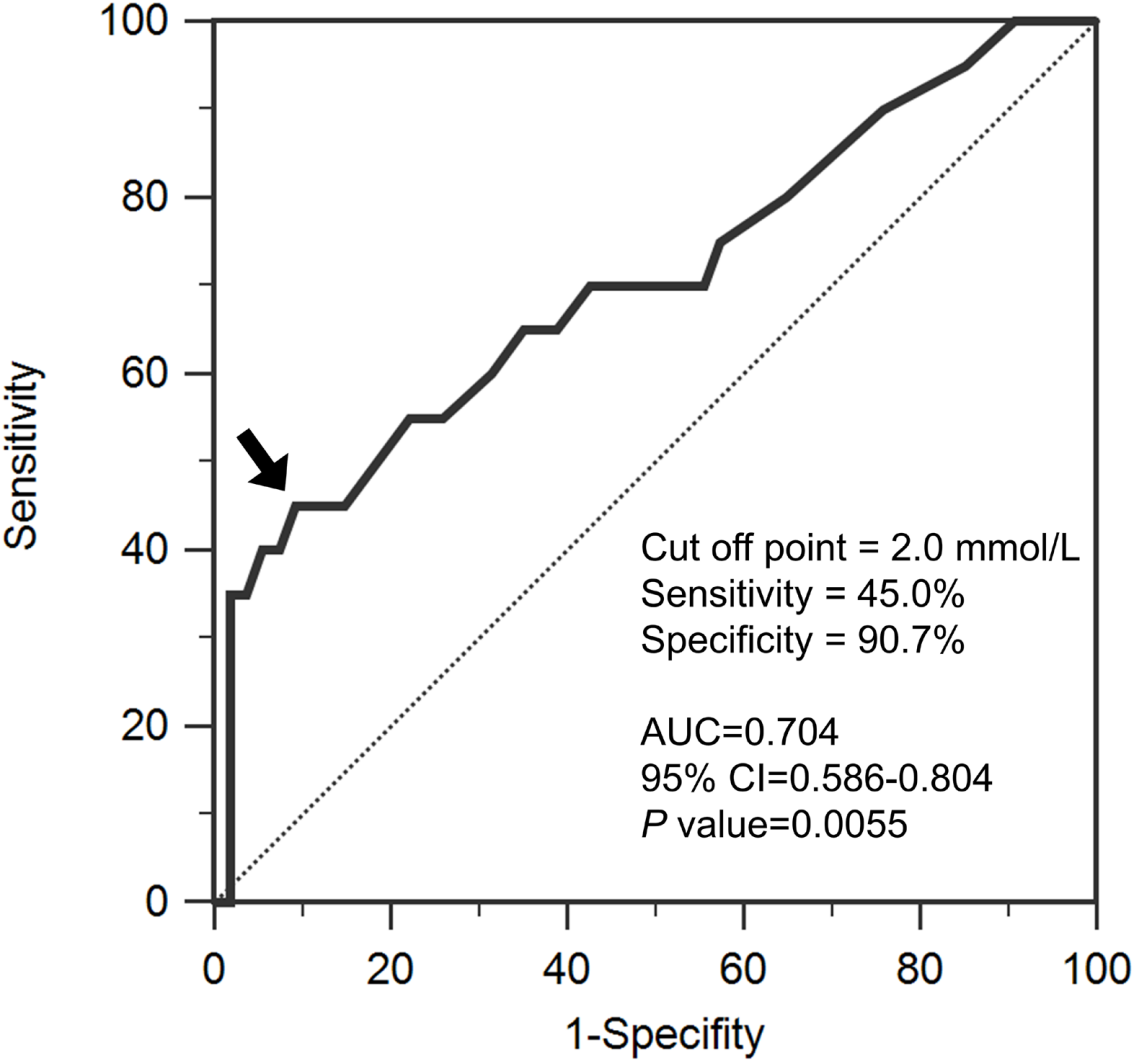
Receiver operating characteristic analysis of serum lactate levels for differentiating between high-grade and low-grade brain tumors The area under the curve (AUC) is 0.705 (95% confidence interval [CI], 0.586–0.804). The arrow indicates the optimal cutoff value for lactate (2.0 mmol/L). The dotted line is the no-discrimination curve.

A total of 74 patients (23 men and 51 women) with solitary supratentorial gliomas were enrolled. Fifty-four patients had low-grade tumors, and 20 patients had high-grade tumors. Table 1 presents the patients’ demographic data. No patients had sepsis or abnormal liver function before surgery. Serum lactate levels were significantly higher in the patients with high-grade brain tumors than in the patients with low-grade brain tumors (1.91 ± 1.06 vs. 1.19 ± 0.78 mmol/L; *p* = 0.011; Table 1). In addition, the patients with high-grade brain tumors tended to have lower serum GFAP than those with low-grade tumors (0.145 ± 0.354 vs. 0.281 ± 0.522 ng/mL; *p* = 0.097; Table 1). Logistic regression analysis revealed that elevated serum lactate levels are an independent predictor of high-grade brain tumors (odds ratio, 2.89; 95% confidence interval [CI], 1.485–2.62; *p* = 0.0018; Table 2]. Receiver operating characteristic (ROC) curve analysis of serum lactate levels was performed to differentiate between high-grade and low-grade brain tumors (Figure 1). The area under the curve (AUC) was 0.705 (95% CI, 0.586–0.804; *p* = 0.0055). Pretreatment serum lactate levels (cutoff value, 2.0 mmol/L) were determined to predict high-grade brain tumors (sensitivity, 45%; specificity, 90.7%).

**Table 1:** Patient characteristics

Characteristic	High grade	Low grade	*P* Value
	(N=20)	(N=54)	
Age (years)	54.2±12.5	57.4±10.9	0.293
Male no. (%)	8 (40.0)	15 (27.8)	0.398
Body weight (kg)	58.9±8.7	60.9±9.9	0.455
**Comorbidities, No. (%)**			
Cardiovascular disease	2 (10.0)	4 (7.4)	0.659
Hypertension	5 (75.0)	9 (16.7)	0.308
Diabetes	3 (15)	4 (7.4)	0.379
Other cancer	1 (5.0)	4 (7.4)	0.589
Tumor size (cm)	4.50±1.78	4.31±1.73	0.450
**WHO grade**			
I/II		38/13	
III/IV	4/16		
**Serum biomarkers**			
Lactate (mmol/L)	1.91±1.06	1.19±0.78	0.011*
LDH (mU/mL)	39.62±31.28	37.70±38.81	0.401
NGAL (mcg/mL)	0.544±0.536	0.465±0.554	0.516
GFAP (ng/L)	0.145±0.354	0.281±0.522	0.097
NSE (mcg/mL)	10.77±14.68	16.86±63.63	0.339
S100β (pg/mL)	93.61±131.76	84.59±129.06	0.353

WHO, World Health Organization; LDH, lactate dehydrogenase; NGAL, neutrophil gelatinase-associated lipocalin; GFAP, glial fibrillary acidic protein; NSE, neuron-specific enolase.

Continuous variables are expressed as the mean ± standard deviation, whereas categorical variables are expressed as the number and percentage.

*p < 0.05.

**Table 2:** Logistic regression analysis of the factors associated with high-grade

Variable	Univariate		Multivariate	
	Odds ratio (CI)	*P*	Odds ratio (CI)	*P*
Age (years)	0.976 (0.933-1.021)	0.291	—	
Sex (male:female)	1.733 (0.592-5.077)	0.316	—	
Body weight (kg)	0.979 (0.926-1.034)	0.438	—	
**Comorbidities**				
Cardiovascular disease	1.389 (0.234-8.240)	0.718	—	
Hypertension	1.667 (0.483-5.751)	0.419	—	
Diabetes	2.206 (0.448-10.87)	0.331	—	
Other cancer	0.658 (0.069-6.267)	0.716	—	
Tumor size (cm)	1.065 (0.793-1.431)	0.675	—	
**Serum biomarkers**				
Lactate (mmol/L)	2.374 (1.275-4.421)	0.006*	2.890 (1.485-2.621)	0.002*
LDH (mU/mL)	1.001 (0.988-1.015)	0.841	—	
NGAL (ng/mL)	1.297 (0.519-3.242)	0.578	—	
GFAP (mg/L)	0.447 (0.095-2.103)	0.308	0.245 (0.050-1.191)	0.083
NSE (ng/mL)	0.997 (0.984-1.011)	0.686	—	
S100β (pg/mL)	1.001 (0.997-1.004)	0.789	—	

Abbreviations: HR, hazard ratio; CI, confidence interval; LDH, lactate dehydrogenase; NGAL, neutrophil gelatinase-associated lipocalin; GFAP, glial fibrillary acidic protein; NSE, neuron-specific enolase.

*p < 0.05.

**Figure 2:**
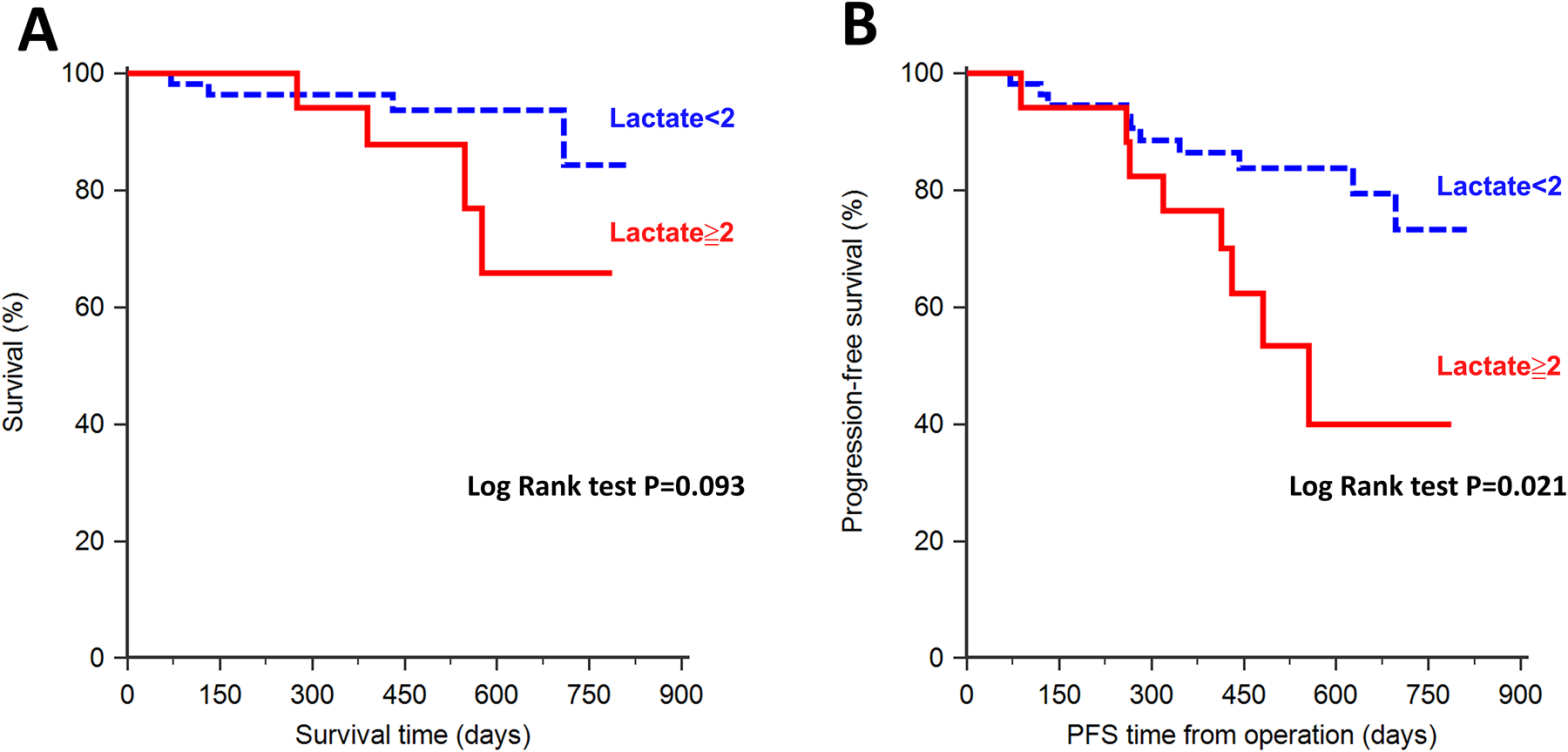
Kaplan–Meier analysis of serum lactate levels and patient survival **(A)** Overall survival (OS) did not differ significantly between patients with high serum lactate levels (≧2.0 mmol/L) and those with low serum lactate levels (*p* = 0.093). **(B)** Progression-free survival (PFS) differed significantly between patients with high serum lactate levels and those with low lactate levels (*p* = 0.021). Patients with high serum lactate levels were associated with poor PFS after tumor resection.

Kaplan–Meier survival analysis revealed a significant relationship between pretreatment serum lactate levels (≧2.0 mmol/L) and oncological outcomes during a follow-up period of 248.5 ± 118.9 days (Figure 2). Patients with higher serum lactate levels had a tendency of shorter overall survival (OS), however, this did not reach statistical significance (Figure 2A; *p* = 0.093). In addition, patients with higher serum lactate was associated with shorter progression-free survival (PFS) than those with lower serum lactate levels (Figure 2B; *p* = 0.021).

Univariate analysis indicated that the male sex and higher serum lactate and NGAL levels are poor prognostic factors (Table 3). However, only the serum lactate level was an independent factor associated with poor OS (HR, 2.049; 95% CI, 1.006–4.174; *p* = 0.048) in a multivariate Cox proportional hazards regression model. High serum lactate levels and the male sex were independently associated with poor PFS (HR, 1.658; 95% CI, 1.078–2.548; *p* = 0.021 and HR, 2.951; 95% CI, 1.161–7.496; *p* = 0.023, respectively).

**Table 3:** Univariate and multivariate Cox regression analyses of overall survival and progression-free survival in patients with supratentorial primary brain tumors

Factor	Progression-free survival					
	Univariate analysis			Multivariate analysis		
	HR	95% CI	*P*	HR	95% CI	*P*
Age (years)	1.029	0.985-1.076	0.195	—	—	—
Sex (male: female)	3.019	1.192-7.645	0.020*	2.951	1.161-7.496	0.023*
Body weight (kg)	1.011	0.965-1.059	0.647	—	—	—
Tumor size (cm)	1.123	0.868-1.453	0.378	—	—	—
**Serum biomarkers**						
Lactate (mmol/L)	1.619	1.079-2.428	0.020*	1.658	1.078-2.548	0.021*
LDH (mU/mL)	1.009	0.999-1.019	0.067	—	—	—
NGAL (ng/mL)	2.062	0.993-4.279	0.052	—	—	—
GFAP (mg/L)	1.260	0.460-3.447	0.653	—	—	—
NSE (ng/mL)	0.998	0.989-1.008	0.740	—	—	—
S100β (pg/mL)	1.001	0.997-1.004	0.733	—	—	—
**Factor**	**Overall survival**					
	**Univariate analysis**			**Multivariate analysis**		
	**HR**	**95% CI**	***P***	**HR**	**95% CI**	***P***
Age (years)	1.041	0.973-1.114	0.247	—	—	—
Sex (male: female)	4.349	1.037-18.247	0.045*	3.785	0.903-15.859	0.069
Body weight (kg)	0.994	0.925-1.070	0.881	—	—	—
Tumor size (cm)	1.214	0.835-1.763	0.310	—	—	—
**Serum biomarkers**						
Lactate (mmol/L)	2.046	1.078-3.886	0.029*	2.049	1.006-4.175	0.048*
LDH (mU/mL)	1.013	0.998-1.028	0.084	—	—	—
NGAL (ng/mL)	2.987	1.067-8.363	0.037*	—	—	—
GFAP (mg/L)	0.983	0.169-5.704	0.985	—	—	—
NSE (ng/mL)	0.936	0.867-1.069	0.478	—	—	—
S100β (pg/mL)	0.997	0.987-1.006	0.490	—	—	—

Abbreviations: HR, hazard ratio; CI, confidence interval; LDH, lactate dehydrogenase; NGAL, neutrophil gelatinase-associated lipocalin; GFAP, glial fibrillary acidic protein; NSE, neuron-specific enolase.

*p < 0.05.

## DISCUSSION

The major finding of this study is that the pretreatment serum lactate level is a more valuable prognostic biomarker than other biomarkers, including neuronal (NSE, S100β, and GFAP) and nonneuronal (NGAL and LDH) biomarkers, in patients with supratentorial primary brain tumors undergoing tumor resection.

The higher pretreatment serum lactate levels in patients with brain tumors may be produced by the metabolic processes of cancer cells and injured normal brain tissues. Cancer cells exhibit a high rate of aerobic glycolysis (Warburg effect), producing lactate that participates in tumor progression [10]. Lactate is not only a nutrient for tumor cells but also a signaling agent that promotes angiogenesis and an immunosuppressive metabolite [11, 12]. Therefore, lactate may be a key regulator of the glycolytic phenotype, which provides nutrients for tumor growth and metastasis. Consequently, massive lactate accumulation is a unique phenotype of cancer [13]. Furthermore, higher lactate levels and poorer patient outcomes have been significantly correlated [14]. For instance, an association between higher lactate levels in tumor tissues and shorter PFS has been widely reported in patients with gliomas [15–17], head and neck cancers [18, 19], and cervical cancer [20]. The present study revealed that the pretreatment serum lactate level is an independent predictor of poor outcomes in patients undergoing brain tumor resection. Because serum lactate levels have a positive linear correlation with tissue lactate levels [21], higher serum lactate levels are possibly representative of higher tissue lactate accumulation.

In addition to indicating the metabolism pathways of cancer cells, elevated baseline serum lactate levels in patients with brain tumors may represent the tumor burden on normal brain tissues. Serum lactate produced by tumor cells is associated with increased LDH levels. A recent meta-analysis showed that higher pretreatment serum LDH levels are associated with poorer oncological outcomes in patients with solid tumors [9]. Although pathologically high pretreatment LDH levels were observed in our study patients, pretreatment LDH levels were not associated with postoperative oncological outcomes, indicating that the association between pretreatment serum lactate levels and oncological outcomes may not be entirely related to tumor metabolism. Moreover, serum lactate accumulates under activated or pathological conditions in the brain, and can be rapidly released into the blood [22]. Therefore, part of the elevated serum lactate level may reflect the tumor burden on normal brain tissues.

Lactate accumulation is commonly elevated in high-grade brain tumors [23]. Our results regarding the association between serum lactate levels and brain tumor grading are consistent with a study by Ramamani et al., with comparable sensitivity (59.3%) and specificity (95.7%) for predicting high-grade brain tumors [8]. Compared with the aforementioned study, the present study not only evaluated more biomarkers but also enrolled more patients (50 vs. 74), and the cutoff value of serum lactate levels was calculated using a more robust statistical approach involving ROC curve analysis and multiple logistic regression. Although our results indicated that pretreatment serum lactate has high specificity in predicting tumor grade, it may be insufficiently sensitive. This may be because serum lactate could be elevated in patients with large, low-grade brain tumors, which have a large tumor burden. The brain tumor grade is most sensitively diagnosed through imaging studies, such as magnetic resonance imaging. Because serum lactate is a convenient biomarker, combining the serum lactate level with imaging tools may assist physicians in clinical management or risk stratification before brain tumor resection.

Our result indicated that male sex may present as a risk factor of worse PFS and a tendency of worse overall survival which is consistent with one study revealing a worse survival after tumor resection surgery in male pediatric brain tumor patients [24]. This may be because the effects of sex are strong in particular subtypes of high-grade glioma. The mesenchymal, proneural, and neural subtypes of glioblastoma multiforme (GBM) exhibit a substantial difference in incidence between male and female patients (2:1) [25]. By contrast, the classical subtype occurs equally in males and females. These differences in cell type may be the reason behind the differences in oncological outcome between male and female patients.

The association between NGAL and solid tumors has received attention in recent years [6]. For instance, urinary NGAL was reported to be associated with brain tumors and decrease with treatment, and can be tracked from brain tumors to urine [5]. Accordingly, we observed that pretreatment serum NGAL levels were markedly elevated in patients with brain tumors. However, pretreatment serum NGAL levels were not associated with oncological outcomes, possibly because NGAL not only participates in tumor development but also exerts anticancer effects [6, 26]. Therefore, the pretreatment serum NGAL level may not be a prognostic biomarker in patients with brain tumors.

In the present study, we observed that certain pretreatment serum neuronal biomarkers, namely NSE, S100β, and GFAP, were not associated with postoperative oncological outcomes. Serum NSE has been widely reported as a tumor marker in diagnosis of brain metastases of small cell lung cancer [27] but not in primary brain tumors, it is possibly because changes in serum NSE levels are sensitive to damages in normal brain tissues caused by tumor compression. Serum S100β has been proposed as an early predictor of brain metastasis [28] and a prognostic factor for brain metastatic melanoma [29]. However, it was not a prognostic biomarker of primary brain tumors, potentially because S100β is less specific to neurons and is distributed in several cell tissues [30]. Several studies have suggested that the serum GFAP level is a potential prognostic factor in patients with primary brain tumors. For instance, GFAP was associated with tumor volume and histopathological tumor characteristics in GBM [4], whereas brain metastases did not increase blood GFAP levels [31]. Pretreatment serum GFAP levels were not significantly associated with postoperative oncological outcomes, possibly because the number of cells expressing GFAP is inversely proportional to the extent of anaplasia, and loss of GFAP expression is frequently observed in high-grade gliomas [32]. Despite GFAP lacking clinical prognostic value, our data are consistent with the aforementioned findings that patients with high-grade brain tumors tend to have lower serum GFAP levels [33].

This study has some limitations that must be addressed in further research. First, the sample size was relatively small; only 54 and 20 patients with low-grade and high-grade brain tumors, respectively, were enrolled. Accordingly, minor risk factors for poorer outcomes, such as low GFAP levels, as well as the lower survival rate in patients with higher serum lactate levels, may not have been detectable. Further investigation in a larger cohort is warranted. Second, only patients with supratentorial primary brain tumors were enrolled because this study was based on the design of an intraoperative fluid management study. Infratentorial brain tumors are typically more difficult to treat than supratentorial brain tumors; therefore, our results may not be completely applicable to patients with infratentorial tumors.

## MATERIALS AND METHODS

### Patients

A total of 74 cases of solitary supratentorial gliomas were retrieved from a patient cohort designed for intraoperative goal-directed fluid therapy at National Taiwan University Hospital (NTUH) during 2013–2015 (NCT02113358). This study was approved by the Ethics Review Committee of NTUH, with written informed consent obtained from all patients. Patients with a body mass index > 27 or < 18.5, congestive heart failure, renal dysfunction, or chronic obstructive pulmonary disease were excluded. In addition, patients with metastatic brain tumors or multiple brain tumors were excluded. Patients did not receive preoperative radiotherapy or chemotherapy.

### Demographic data, imaging, and pathology

Data on patient demographics, comorbidities, tumor histology, and World Health Organization (WHO) tumor grading were analyzed. Tumors were classified as high-grade (WHO grade III, IV) or low-grade (WHO grade I, II). Tumor sizes were measured based on the longest tumor diameter in magnetic resonance imaging in a single axial section.

OS was calculated from the date of initial tumor resection to patient death. PFS was determined from the date of initial tumor resection to tumor recurrence or patient death.

### Serum biomarker assessment

Levels of serum neuronal and nonneuronal biomarkers were assessed before tumor resection. Levels of the neuronal biomarkers, namely NSE (Alpha Diagnostic International, San Antonio, TX, USA), S100β (BioVendor LLC, Candler, NC, USA), and GFAP (BioVendor LLC, USA) were measured using enzyme-linked immunosorbent assay kits. Lactate levels were quantified using a blood gas analyzer (Stat Profile Critical Care Xpress, Nova Biomedical Cooperation, Waltham, MA, USA). LDH activity was assessed using an activity assay kit (Biovision, Milpitas, CA, USA).

### Statistical analysis

The Student t test and Mann–Whitney U test were used for variables with a parametric distribution and a nonparametric distribution, respectively. The χ^2^ test or Fisher exact test was used for assessing the statistical significance of categorical variables. To identify factors associated with high-grade brain tumors, patient age, sex, body weight, comorbidities, and serum biomarkers (lactate, LDH, NGAL, GFAP, NSE, and S100β) were included in the univariate and multivariate logistic regression analyses. Age, body weight, and serum biomarkers were used as continuous variables, and the remaining covariates were used as categorical variables.

A ROC curve was constructed to analyze independent predictors of tumor grading and to estimate the AUC, sensitivity, and specificity of the optimal cutoff value (calculated using Youden index).

OS and PFS were evaluated using the Kaplan–Meier method for univariate analysis, and differences were assessed using the log-rank test. Univariate and multivariate Cox proportional hazards regression models were used to investigate the effects of different variables on OS. Data were presented as the mean ± standard deviation, and data were statistically analyzed using SPSS V20.0 software. Values of *p* < 0.05 were considered statistically significant, and all *p* values reported were two-sided.

## CONCLUSION

This study suggests that an elevated pretreatment serum lactate level is an independent predictor of poorer oncological outcomes, including shorter OS and PFS, and is associated with higher tumor grades in patients undergoing supratentorial primary brain tumor resection. By contrast, pretreatment levels of neuronal biomarkers, namely S100β, NSE, and GFAP, and nonneuronal biomarkers, namely NGAL and LDH, were not associated with oncological outcomes.

## Abbreviations

HR: hazard ratio
CI: confidence interval
NSE: neuron-specific enolase
GFAP: glial fibrillary acidic protein
NGAL: neutrophil gelatinase-associated lipocalin
OR: odds ratio
ROC: receiver operating characteristic
OS: overall survival
PFS: progression-free survival
LDH: lactate dehydrogenase
GBM: glioblastoma multiforme
WHO: World Health Organization
AUC: area under the curve
